# Prevalence and risk factors for *Salmonella* spp. colonization in broiler flocks in Shiraz, southern Iran

**Published:** 2014

**Authors:** Maryam Ansari-Lari, Shahram Shekarforoush, Samira Mehrshad, Hosna Safari

**Affiliations:** *Department of Food Hygiene and Public Health, School of Veterinary Medicine, Shiraz University, Shiraz, Iran.*

**Keywords:** Antibiotic, Broiler flocks, Iran, *Salmonella*, Vaccination

## Abstract

*Salmonella* spp. are important food borne pathogens worldwide that frequently infect poultry flocks. This cross-sectional study was conducted to determine the prevalence of *Salmonella* spp. colonization in broiler flocks in Shiraz (southern Iran) and to find the possible association of infection status with some potential risk factors including vaccination program and use of antibiotics. During October 2009 to April 2010, a total of 40 broiler flocks were selected in slaughterhouse and 20 cloacae contents were collected from each flock. Every five cloacae contents were pooled and investigated for *Salmonella* spp. using appropriate culture methods. The flock was considered positive if any of the pooled samples turned positive in culture. Statistical analysis was performed using multiple logistic regression. Nine out of 40 flocks (22.50%, 95% CI: 9-36) were positive for *Salmonella* spp. colonization. Nearly 75.00% of flock owners reported that they used antibiotics during production period, more frequently fluoroquinolones, combination of trimethoprim-sulfonamides (TMP/SU) and tetracycline. Nearly 60.00% of the flocks which had used TMP/SU were positive for *Salmonella* spp. compared with 10.00% of the flocks which did not use this antibiotic (*p* = 0.006). Increasing flock age was associated with a decreased chance of *Salmonella* spp. detection (*p* = 0.003). In flocks which received infectious bronchitis vaccine, 36.00% were positive for *Salmonella* spp. whereas this was 15.00% for flocks which did not receive this vaccine (*p* = 0.08). Careful monitoring of antibiotics use and further studies to determine the most appropriate vaccination program in the field is recommended.

## Introduction

*Salmonella* spp. are important food borne pathogens worldwide that frequently infect poultry flocks. Poultry become infected with *Salmonella *spp. by direct contact with infected birds, consumption of contaminated feed or water and through the environment.^1^ Contamination of poultry and poultry products with *Salmonella* spp. seems to be mostly linked to flock colonization.^2^^,^^3^ Consumption of raw or undercooked contaminated poultry products can induce acute gastroenteritis in humans and reducing the prevalence of colonization at poultry flocks is likely to reduce the risk of human salmonellosis from broiler chicken consumption. 

The *Salmonella* spp. prevalence in broiler flocks varied considerably between countries, nearly 0% in Sweden and 68.20% in Hungary,^4^ 76.90% in Canada,^5^ 69.80% in France,^6^ 41.30% in Turkey,^7^ and 25.00% in Denmark.^8^

Several management and environmental risk factors have been shown to be associated with *Salmonella* spp. infection of the flocks. In epidemiological studies, inadequate cleaning and disinfection have been reported as important risks for *Salmonella *spp. persistence in poultry houses.^3^^,^^6^^,^^9^^-^^11^ Also, control of rodents and insects between production periods is an important factor for reducing *Salmonella* spp. infection.^9^^,^^12^ However, there is limited information about the association of *Salmonella* spp. with vaccination program (for infections other than salmonella) and using antibiotics in broiler flocks. 

There are several studies concerning isolation, characterization, and prevalence of *Salmonella* spp. from poultry carcasses, poultry meat and broiler flocks in Iran.^13^^-^^16^ However, there is no epidemiologic study investigating the prevalence and flock level risk factors of salmonella in broilers in Iran. Therefore, in the present study we aimed at determining the prevalence of *Salmonella* spp. colonization in broiler flocks in Shiraz (southern Iran) and its possible associations with some less investigated risk factors including vaccination program and use of various types of antibiotics during the production period. 

## Materials and Methods

This is a cross-sectional study which was conducted in Shiraz, the capital of Fars province in southern Iran. During October 2009 to April 2010, a total of 40 broiler flocks were selected at the time of slaughtering and 20 cloacae contents were collected from each flock in poultry slaughterhouses. Every five cloacae contents were pooled and approximately 1 g of pooled samples was inoculated into the lactose broth (Merck, Darmstadt, Germany) before being incubated at 37 ˚C for 24 hr. One mL of the lactose broth was then used to inoculate into 10 mL of cystine-selenite (Merck, Darmstadt, Germany) and another 0.1 mL was used to inoculate into Rappaport Vassiliadis broth (RV broth; Merck, Darmstadt, Germany). The latter cultures were incubated for 18- 24 hr at 37 ˚C and 41.5 ˚C. Loop full inoculums were subsequently streaked on Salmonella-Shigella agar (SS agar; Merck, Darmstadt, Germany) and xylose lysine deoxycholate agar (XLD agar; Merck, Darmstadt, Germany). After incubation at 37 ˚C for 24 hr, the suspected colonies streaked on Brilliant Green agar (BG agar; Merck, Darmstadt, Germany) and the isolates were identified based on their colonies appearance, Gram stain, Triple Sugar Iron agar TSI, Indol, methyl red, Voges-Proskauer and Citrate tests (IMViC), urease, Lysin and hydrolyzes the *ortho*-Nitrophenyl-β-galactoside (ONPG) tests. The flock was considered positive if any of the four pooled samples turned positive in culture as described by Arsenault *et al*.^17^

Data about age and weight at the time of slaughter, flock size, use and type of antibiotics and vaccination program were collected by interviewing the farm owners. Data about cumulative mortality was available for 21 out of 40 flocks. Prevalence of infection and corresponding 95% confidence interval (CI) was estimated. Possible association of *Salmonella* spp. colonization status of the flock with risk factors was investigated using uni-variable and multi-variable logistic regression analysis. Variables with *p*-value equal or less than 0.2 were included in the multivariable logistic regression analysis. Final logistic model was fitted based on stepwise backward elimination procedure and significance of Wald statistics. Data were analyzed using SPSS statistical software (Version 16.0; SPSS, Inc., Chicago, USA) and a *p*-value less than 0.05 was considered statistically significant in the final model.

## Results

Overall, nine out of 40 flocks (22.50%, 95% CI: 9-36) were positive for *Salmonella *spp. Average (± SD) age and weight of flocks at the time of slaughter was 52 ± 5 days and 2370 ± 216 g, respectively. Average cumulative mortality was 8.30%. Summary statistics for flocks attributes according to *Salmonella* spp. colonization status of the flocks are presented in Table 1. The mean weight of the positive flocks (2320 ± 164 g) was slightly lower than the negative ones (2390 ± 229 g); however, the difference was not statistically significant (*p* = 0.43). Nearly 75.00% of flock owners reported that they used one or more antibiotics during production period, more frequently fluoroquinolones, combination of trimethoprim-sulfonamides (TMP/SU) and tetracycline.

Based on uni-variable analysis, among those farms which had reported using antibiotics during production period, TMP/SU showed association with *Salmonella* spp. colonization (*p* = 0.003); nearly 60.00% of the flocks which had used TMP/SU were positive for *Salmonella* spp. compared with 10.00% of the flocks which did not use this antibiotic. Using two or more type of antibiotics did not show significant association with *Salmonella *spp. colonization of the flock (*p* = 0.21).

**Table 1:** Summary statistics for flocks attributes according to *Salmonella* status in 40 broiler flocks from Shiraz at slaughter, southern Iran. Data are presented as mean ± SD

**Attribute**	**Positive flocks** **(n = 9)**	**Negative flocks** **(n = 31)**	***p*** **-value**
**Weight (g)**	2322 ± 164	2388 ± 229	0.43
**Age (day)**	49.70 ± 5.30	52.40 ± 4.40	0.20
**Flock size (n)**	28389 ± 18327	21746 ± 11569	0.13
**Mortality (%)**	8.90 ± 7.50	8.10 ± 4.20	0.79

**Table 2:** Multivariable logistic regression analysis of risk factors for *Salmonella* in 40 broiler flocks from Shiraz, southern Iran

**Attribute**	**Wald **	**Standard error**	***p-value***	**Odds ratio**
**Trimethoprim sulfonamides**				
**Yes**	7.60	1.36	0.006	43.10
**No** *	-	-	-	1.00
**Age (day)**	8.70	0.02	0.003	0.93
**Infectious bronchitis vaccine**				
**Yes**	3.10	1.25	0.080	8.90
No*	-	-	-	1.00

* Reference category.

All the flocks received Newcastle and infectious bursal disease vaccines; however, only 35.00% received infectious bronchitis (IB) vaccine. In flocks which received IB vaccines, 36.00% were positive for *Salmonella *spp. whereas this measure was 15.00% for flocks which did not receive this vaccine (*p* = 0.15).

Overall, four variables including using TMP/SU (*p* = 0.003), IB vaccination (*p* = 0.15), age at slaughter (*p* = 0.13) and flock size (*p* = 0.20) were included in the logistic model. Through stepwise backward elimination method with excluding the intercept, three variables were remained in the final model (Table 2). Results showed that odds of infection decreased with increasing age of the flock (*p* = 0.04) and increased in the case of using TMP/SU (*p *= 0.007) and IB vaccination (*p* = 0.08). 

## Discussion

The prevalence of *Salmonella* spp. in the flocks in the present study was 22.50% which is comparable with some European countries such as Ireland (27.00%),^18^ and Denmark (25.00%).^8^ However, possibility of false negative results and underestimation of the prevalence of *Salmonella* spp. could not be eliminated because sensitivity of microbiological methods used to isolate *Salmonella* spp. is limited**.**

Nearly 75.00% of the flock owners reported that they use various types of antibiotics during the production period, more frequently fluoroquinolones, combination of TMP/SU and tetracycline. Many poultry producers use antibiotics for growth promotion and/or disease prophylaxis and treatment. Field studies concerning associations between using antibiotics and *Salmonella* spp. prevalence in poultry flocks are very limited and indicated that using antibiotics is associated with decreasing prevalence of *Salmonella* spp. in poultry flocks.^3^^,^^19^ This is in contrast to the finding of the present study which showed that using TMP/SU in the flocks increased the odds of *Salmonella *spp. Due to possibility of confounding effect of other management practices on the observed association, flock age, mean weight at slaughter and flock size were compared between flocks with and without use of TMP/SU. No significant difference was detected (data not shown). Resistance of *Salmonella* spp. to TMP/SU has been reported in an *in vitro* study^20^ and in a study by Dallal *et al*.^13^ Taken together, it could be concluded that this antibiotic may not be a good choice for widespread use in poultry flocks; and use of antibiotics in the poultry farms needs careful monitoring.

Significant negative association was observed between infection status of the flock and age at slaughter. Vertical transmission of *Salmonella* spp. from parent flocks may be a possible explanation for this observation. This is in contrast to the results for another important food borne pathogen, campylobacter, which had been shown to have positive association with age of the flock.^21^

Association between IB vaccination and *Salmonella* spp. was nearly significant in our final model (*p* = 0.08) which could be attributed to the small sample size in our study. Although IB vaccination is used in a nationwide program in the country, significant proportion of farm owners did not use IB vaccination in their flocks due to their undesirable personal experience with this vaccine in the field.^22^ Therefore, we had the opportunity to evaluate the association of the IB vaccine and *Salmonella* spp. in broiler flocks. To the best knowledge of authors, the association of *Salmonella* spp. with IB vaccination in broiler flocks has not been addressed previously. The only exception is a recent study by Volkova *et al*. which investigated the association between vaccination against protozoal and viral infections and *Salmonella* spp. in broiler flocks.^23^ They indicated that increased dosage of IB viral vaccine delivered via spraying to the 1-day-old birds was linked to a higher probability of detecting *Salmonella* spp. in the flock during rearing and on the broiler carcasses at the pre-chilling and post-chilling points in processing,^23^ which is in agreement with our finding in the present study. Before providing any explanation for this observation, future studies with larger sample size in this context are warranted. 

In conclusion, the overall prevalence of *Salmonella* spp. colonization was 22.50% in our studied flocks. Age at slaughter, TMP/SU antibiotics and IB vaccination were associated with *Salmonella* spp. colonization of the flock. Careful monitoring of using antibiotics and further studies to determine the most appropriate vaccination program in the field is recommended. 
